# Enterobius Vermicularis Induced Bartholin Gland Abscess

**DOI:** 10.5146/tjpath.2024.13381

**Published:** 2025-09-30

**Authors:** Saadia Makni, Manel Mellouli, Mouna Zghal, Lobna Bouzidi, Slim Charfi, Tahya Sellami Boudawara, Marwa Bouhamed

**Affiliations:** Department of Pathology, Habib Bourguiba University Hospital, Sfax, Tunisia

**Keywords:** Bartholin gland, Abscess, Enterobius vermicularis, Pinworm, Oxyuriasis

## Abstract

Bartholin gland abscesses are typically caused by bacterial agents. Abscesses induced by Enterobius vermicularis are exceptional. We report, here, the case of a 27-year-old woman, whose histopathological examination of the Bartholin gland cyst confirmed the presence of E. vermicularis eggs in the lumen of the cyst.

## INTRODUCTION

Enterobiasis is a common parasitic condition in the world, especially in temperate climate countries. It is caused by Enterobius vermicularis (pinworm), which is a helminth belonging to the nematode family. The parasites affect mainly children and most infections are asymptomatic. Man will be contaminated by ingesting E. vermicularis eggs that colonize the intestinal lumen. Extraintestinal locations by ectopic migration of the parasite are rare and the female genital tract is the most common site (1). Enterobiasis has been reported in the vaginal wall, cervix, endometrium, ovary, and fallopian tube (2,3). Bartholin gland affection is extremely rare (4,5). We report here a case of Bartholin gland abscess caused by E. vermicularis eggs to clarify the etiopathogenic particularities and to describe the clinical and pathological criteria of this rare pathology.

## CASE REPORT

**Figure 1:**
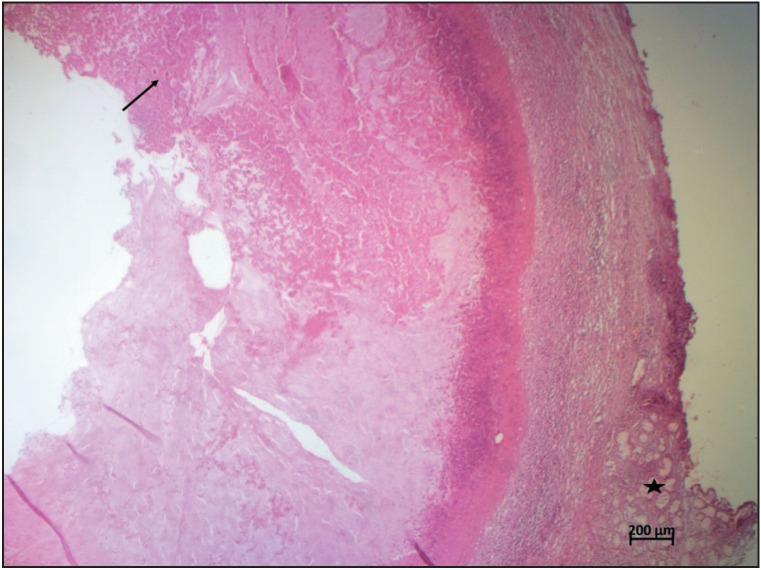
A cystic wall containing mucous Bartholin gland (asterisks) bordered by inflammatory infiltrate. The lumen contained necrotic material with oval-shaped, asymmetrical parasite eggs (arrow) (H&E×25)

**Figure 2:**
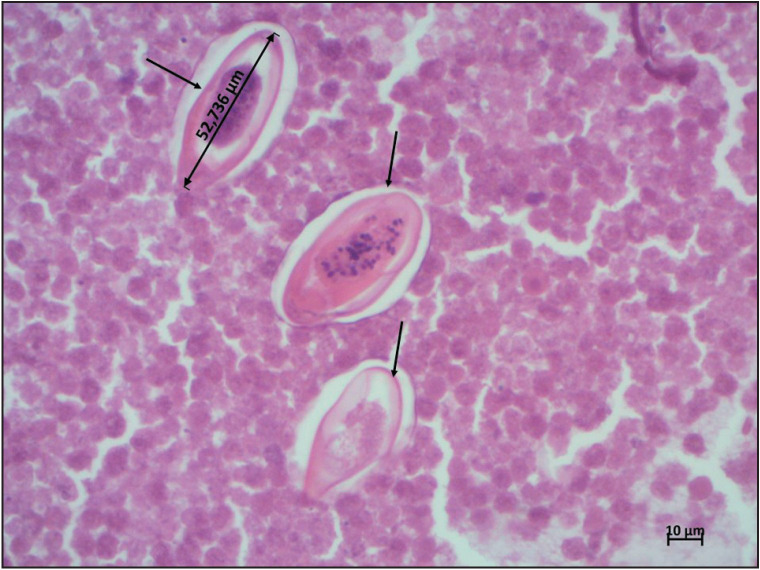
The egg of Enterobius vermicularis at high magnification (arrows) (H&E×400).

A 27-year-old woman, with no past medical or surgical history, presented with a vulvar mass. According to the patient, this mass had been present for more than six months. On gynecological examination, it was a cystic mass, painless on palpation, primarily suggestive of a Bartholin gland cyst. A surgical excision was done and the patient received antibiotherapy based on Levofloxacin and Clindamycin. The histopathological analysis found a cystic wall containing mucous Bartholin gland (Figure 1, asterisks) bordered by inflammatory infiltrate consisting of macrophages, lymphocytes, neutrophils, and eosinophils. The lumen contained necrotic material with oval-shaped, asymmetrical parasite eggs, which were approximately 52 µm. This size and appearance were typical for *Enterobius Vermicularis *eggs (Figure 1, Figure 2, arrows). Upon retrospective questioning, the patient revealed recurrent episodes of anal and vulval itching exacerbated at night as well as a personal and family history of intestinal pinworm disease. The Scotch tape test was performed after obtaining the histopathology report and the stool examination was positive for the eggs and adult worm of E. vermicularis. Treatment with Mebendazole 100mg was initiated for the patient and all household members. The patient was well with no recurrence at five months of follow-up.

## DISCUSSION

Bartholin gland abscesses are typically caused by obstruction of the Bartholin gland duct, leading to an accumulation of fluid and subsequent infection. The most common pathogens associated with Bartholin’s gland abscesses are bacteria, particularly those from the gastrointestinal tract such as Escherichia coli, and sexually transmitted pathogens like Chlamydia and Neisseria (1). However, in rare instances, various other organisms can be implicated. In the English literature, only one case report of a 45-year-old woman has been documented, in which E. vermicularis eggs were detected in the aspirate of the Bartholin gland abscess (4).

Regarding etiopathogenesis, there is no well-established direct link between oxyuriasis and Bartholin gland abscesses. However, it is conceivable that if oxyuriasis leads to severe perineal itching, excessive scratching could potentially introduce bacteria from the perianal region into the Bartholin’s gland duct, increasing the risk of infection (4,5).

On clinical examination, at this site, the parasite often causes an inflammatory mass or a pseudo-tumoral granuloma (1–4).

The differential diagnosis includes various parasitic infections, namely Entamoeba histolytica, Microfilaria, Strongyloides stercoralis, Schistosoma haematobium, Trichuris trichiura, Ascaris, and Taenia (6). To establish a diagnosis, it is crucial to integrate clinical findings with laboratory investigations like stool examination and culture. Under the microscope, these parasites and their eggs can be differentiated based on distinct morphological features.

Adult female worms found in tissue sections have a maximum diameter of 500 μm while males reach up to 200 μm. Both sexes show a muscular wall and exhibit prominent lateral alae.

Enterobius vermicularis eggs measure approximately 50 to 60 μm in length and 20 to 30 μm in width. These eggs typically possess a thick shell, flattened on one side, and contain a larva within.

Treatment consists of the abscess drainage and administration of antiparasitic medications such as mebendazole or albendazole to eradicate the E. vermicularis infection. Additionally, antibiotics may be used to treat any associated bacterial infection (2–5).

In conclusion, E. vermicularis or pinworm is an exceptional cause of Bartholin gland abscesses. This article highlights the importance of considering parasitic infections as a possible etiology in patients presenting with gynecological symptoms. Further research and awareness are needed to better understand the pathogenesis, diagnosis, and management of such cases.

## Ethical Approval

The authors certify that they have obtained all appropriate patient consent forms. In the form, the patient has given his consent for her clinical information to be reported in the journal.

## Conflict of Interest

No financial or personal interests.

## Funding

No funding received.
